# Identifying meaningful subpopulation segments among older public assistance recipients: a mixed methods study to develop tailor-made health and welfare interventions

**DOI:** 10.1186/s12939-023-01959-7

**Published:** 2023-08-03

**Authors:** Keiko Ueno, Daisuke Nishioka, Junko Saito, Shiho Kino, Naoki Kondo

**Affiliations:** 1 Department of Social Epidemiology, Graduate School of Medicine, School of Public Health, Kyoto University, Kyoto, Japan; 2 Department of Health and Social Behavior, Graduate School of Medicine, The University of Tokyo, Tokyo, Japan; 3 Department of Medical Statistics, Research & Development Center, Osaka Medical and Pharmaceutical University, Osaka, Japan; 4 Division of Behavioral Sciences, National Cancer Center Institute for Cancer Control, Tokyo, Japan; 5 Department of Oral Health Promotion, Graduate School of Medical and Dental Sciences, Tokyo Medical and Dental University, Tokyo, Japan; 6 Institute for Future Initiatives, The University of Tokyo, Tokyo, Japan; 7 Japan Agency for Gerontological Evaluation Study (JAGES Agency), Tokyo, Japan

**Keywords:** Public assistance recipients, Segmentation, Tailored support intervention, Soft clustering, Mixed methods study

## Abstract

**Background:**

Public assistance recipients have diverse and complex needs for health and social support in addition to financial support. Segmentation, which means dividing the population into subgroups (segments) with similar sociodemographic characteristics, is a useful approach for allocating support resources to the targeted segments. Clustering is a commonly used statistical method of segmentation in a data-driven marketing approach. This explanatory sequential mixed methods study applied a clustering technique, aiming to identify segments among older public assistance recipients quantitatively, and assess the meaningfulness of the identified segments in consultation and support activities for older recipients qualitatively.

**Methods:**

We identified the segments of older recipients in two municipalities using probabilistic latent semantic analysis, a machine learning-based soft clustering method. Semi-structured interviews were subsequently conducted with caseworkers to ask whether the identified segments could be meaningful for them in practice and to provide a reason if they could not think of any older recipients from the segment.

**Results:**

A total of 3,165 older people on public assistance were included in the analysis. Five distinct segments of older recipients were identified for each sex from 1,483 men and 1,682 women. The qualitative findings suggested most of identified segments reflected older recipients in practice, especially two of them: female Cluster 1 (facility residents aged over 85 years with disability/psychiatric disorder), and female Cluster 2 (workers). Some caseworkers, however, did not recall older recipients in practice when working with certain segments.

**Conclusions:**

A clustering technique can be useful to identify the meaningful segments among older recipients and can potentially discover previously unrecognized segments that may not emerge through regular consultation practices followed by caseworkers. Future research should investigate whether tailored support interventions for these identified segments are effective.

**Supplementary Information:**

The online version contains supplementary material available at 10.1186/s12939-023-01959-7.

## Background

There is an urgent call for community-based efforts to close the health gap [1]. The approaches to reduce the health gap include improving daily living conditions via equitable redistribution of socioeconomic resources [2]. In many countries, the public assistance program plays an important role in financial redistribution, ensuring the minimum standard of living for people in poverty. Recent studies have reported that despite the financial aid, the health status of public assistance recipients is not as good as that of non-recipients. A systematic review of studies from high-income countries has shown that public assistance recipients have more psychological symptoms, mental disorders, diabetes, and a higher mortality rate than non-recipients [3]. Moreover, recent Japanese studies have revealed the health inequality among people receiving public assistance: for example, those living alone, unemployed, and having foreign nationalities may be linked to poor health, including diabetes, allergic diseases, dental diseases, and unfavorable health behaviors [4–7]. These findings suggest that there is a need for additional non-financial and tailored support among public assistance recipients, such as social interaction support, job seeking activity, and health counseling, based on the individual sociodemographic characteristics and other personal traits.

Compared to younger generations, older people are more likely to have support needs for healthcare, long-term care, daily supports, and social care [8]. In Japan, approximately 1.6% of the population is currently enrolled in the public assistance program (*seikatsu-hogo* in Japanese). Public assistance is a welfare program offered by the government to households that live below the poverty line and have no assets. The municipal welfare office evaluates individuals’ qualification for public assistance by conducting a thorough means test, which examines their personal assets, ability to work, financial assistance received from family members, and utilization of other social programs. In addition to full exemptions from payments for medical and long-term care, eligible households can receive monthly income assistance. The share of household of people aged 65 years or older on public assistance increased from 32.5% to 1985 to 55.1% in 2021 due to the rapidest unprecedented pace of aging population and lingering macroeconomic stagnation since the 1990s [9, 10]. Healthcare support program has been implemented since January 2021 to deal with diverse and complex needs of older public assistance recipients for health and social support [11]. This program is mandated for municipal welfare offices for promotion of disease prevention, maintenance of physical and mental functioning, and promotion of proper use of public assistance. One of the major challenges in implementing this program is to prioritize the intervention targets and care planning [12]. Another obstacle is the arduous task faced by caseworkers in monitoring the health condition, changes, and regular medical appointments of the recipients under their responsibility. Collaborating with healthcare professionals in a timely manner poses a challenge, despite the fact that caseworkers are often the first to identify the health issues of the recipients. Caseworkers are public officers who work in municipal welfare offices. Their duties encompass two primary categories: administrative tasks involving processing paperwork associated with protection applications and the provision of public assistance, as well as interpersonal support through regular home visits and interviews conducted with public assistance recipients. Due to the typical practice of transferring caseworkers between municipal government departments every 3–5 years, the workforce consists of both experienced and novice individuals engaged in assistance activities. Thus, external resources and further innovation are required in order to identify the most appropriate targets for interventions and support measures.

As a means of providing tailored support interventions to targeted people based on their needs, we focused on the theories and practices in business and social marketing [13, 14]. In marketing, the intervention starts from identifying the “audience”: specific individuals/populations to be targeted, and then creating a “marketing mix”: product, price, place, and promotion of the services to be provided. A marketing mix is designed so that it suits the characteristics and interests of the identified audience. Segmentation is the practice of classifying a whole target group into subgroups (segments) based on their characteristics and it is essential for identifying segments of the audience. A segment consists of the persons with similar characteristics [15]. In theory, providing tailored support for each segment is an effective approach to meet their distinct needs [13]. Cluster analysis is a commonly used statistical method for segmentation and it is further classified into soft clustering and hard clustering [16]. Soft clustering allows individuals to belong to multiple segments simultaneously, with specific probabilities to a particular segment, while in hard clustering, individuals can only belong to a single segment [17, 18].

Hence, the objective of this study was to employ marketing segmentation and a soft clustering technique to characterize different segments within the population of older public assistance recipients in Japan. Additionally, the study aimed to evaluate the meaningfulness of these segments for caseworkers by employing a qualitative research approach.

## Methods

### Study design

An explanatory sequential mixed methods design was used [19]. This study consisted of two phases: a quantitative phase identifying the segments of older recipients and a qualitative phase involving semi-structured interviews with caseworkers.

### Quantitative phase

#### Data

We used cross-sectional data of public assistance recipients aged 65 years or older from two suburban municipalities (A and B) in Japan as of January 2016. Using snowball sampling, we recruited these municipalities interested in improving caseworkers’ work efficiency through a company that provided administrative software for the public assistance database. In 2016, municipality A had a population of 100,934 individuals. Among them, 24.8% were aged 65 or older, and 1.80% of the population were public assistance recipients. In contrast, municipality B had a population of 180,277, with 23.9% being aged 65 or older, and 2.8% of the population receiving public assistance. We used data from the public assistance database of municipalities’ welfare offices, which included information on age, sex, household composition, nationality, working status, work income, pension, housing type, reasons for starting public assistance, and type of disability or disease. The data were acquired by personnel working in the welfare offices of respective municipalities during the process of evaluating applications for public assistance. The database included all public assistance recipients in the municipality as of January 2016. We also used the long-term care assistance claims database including information on health and the levels of care needs, based on their physical and cognitive functions, and long-term care service use history. These databases were merged using personal identification numbers. All variables were used as categorical variables (Additional file 1: Table S1).

#### Statistical analysis

Descriptive statistics were used to summarize participants’ baseline characteristics. The clusters of older recipients were identified using probabilistic latent semantic analysis (PLSA) [20]. This soft clustering method assigns two columns of data (individuals and variables) to each cluster and the degree of affiliation with the cluster is given by probability. We assumed that individual (*i*) is characterized by variable (*v*) via latent variable *z* and defined its joint probability by the following equation;

P(_*ii*_,_*vj*_)= $\sum _{k=1}^{K}P$(_*zk*_)P(_*vi*_|_*zk*_)P(_*ij*_|_*zk*_).

P(*z*), P(*v*|*z*), and P(*i*|*z*) were calculated by the EM (Expectation Maximization) algorithm, which maximizes the log likelihood function [21]. This method provides the flexibility of both individuals and variables belonging to more than one cluster.

In this study, the number of clusters was increased from 2 to 10 by changing the initial value five times. The optimal number of clusters cannot be determined by a single criterion in soft clustering methods [22]; thus, the optimal number of clusters was determined based on Akaike information criterion (AIC) [23], Bayesian information criterion (BIC) [24], size of clusters [22], and interpretability of clusters [25]. A variable with a higher affiliation probability to a cluster represents the cluster characteristics [26]; however, there is no fixed value for “high” affiliation probability. Therefore, in this study, we used variables with an affiliation probability of 0.55 or higher, to interpret and describe the cluster characteristics because if the affiliation probability threshold for a variable that characterizes a cluster is set to 0.50, the variable may belong to another cluster with the remaining 0.50 probability.

The analyses were stratified by sex because socioeconomic factors, such as income and occupation, differ according to gender, as determined historically, culturally, and socially [27]. PLSA was performed using Target Finder (Tokyu Agency Inc., Tokyo, Japan), and other statistical analyses were performed using STATA SE V.16.2 (Stata Corp., College Station, Texas, USA).

### Qualitative phase

#### Data

Purposive sampling was used to select interviewees from experienced caseworkers with at least 3 years of experience working in the municipality welfare offices that provided the data for the quantitative study [28, 29]. It is crucial to identify “meaningful” segments when utilizing a data-driven segmentation method. These segments should align with real-world characteristics and resonate with the individuals who will utilize them [14]. Thus, we selected caseworkers with experience with public assistance recipients who would most likely use the resulting segments in the future as interviewees. From September to October 2021, the first author (a female physician with clinical experience of 20 years, who was trained as a qualitative researcher) conducted semi-structured joint interviews with two caseworkers from each welfare office by video conference via Zoom (Zoom Video Communications Inc., U.S.A). A joint interview is an interview format that is between an individual interview and focus groups. During individual interviews, only one interviewee is present, which may result in limited expression or restriction of their statements. In a focus group, there exists the possibility that certain participants may actively contribute or express their opinions [29]. In a joint interview, participants who already have an existing relationship with each other engage in conversation and can elicit statements. However, joint interviews can potentially lead to conflicts between participants under certain conditions. Despite this, we chose to conduct joint interviews because they have the potential to overcome the limitations of individual interviews and focus groups [30]. Semi-structured interview guide was employed during the interviews (Additional file 2). Based on the validity of mixed methods research [31], we defined meaningful segments as the segments from which caseworkers can recall public assistance recipients in their routine consultation and support activities. Therefore, caseworkers were asked whether they could think of older recipients with similar characteristics of each cluster by being shown the clusters obtained in the quantitative phase. Next, they were asked to describe additional attributes of older recipients. They were asked to provide a reason if they could not think of any older recipients from the cluster. Confidentiality and privacy were ensured for each interviewee during the interview.

#### Data analysis

All interviews were recorded, first transcribed in Japanese, and then translated into English. Data analysis was conducted by two researchers (UK and NK) through the qualitative descriptive method described by Yin [29]. After reading the verbatim transcripts, we divided the text into segments. We assigned code labels to these segments and created an emerging set of codes. Subsequently, we organized them into themes. To establish credibility and trustworthiness, the transcribed data and themes were discussed and reviewed among the authors throughout the process. We also mailed a copy of the findings to ask the participants to check for any discrepancies with the intended content.

### Mixed methods integration

Joint display, which showed the integration of quantitative and qualitative findings in a single table, matrix, or figure, was created in order to compare the integrated findings of these results [32, 33]. Integrated results were discussed among researchers by looking at the “fit” of the quantitative and qualitative findings [34]. We interpreted an integrated result as “concordance” (all the interviewees could think of older recipients from the cluster), “partial concordance” (some of the interviewees could think of older recipients from the cluster and others could not), and “discordance” (none of the interviewees could think of older recipients from the cluster).

## Results

### Quantitative findings

A total of 3,165 older people on public assistance were included in the analysis. A high proportion of participants, both males and females, lived alone, were Japanese, did not work, and did not earn working income (Table 1). There were no missing values because the data were collected when applying for public assistance and determining households’ monthly minimum living expenses.

**Table 1:** Characteristics of public assistance recipients aged 65 years or older stratified by sex　(n = 3,165)

		Male (n = 1,483)	Female (n = 1,682)
Variables		**n (%)**	**n (%)**
Age (years)	65 − 74	905(61.0)	734(43.6)
	75 – 84	513(34.6)	698(41.5)
	85 and over	65(4.4)	250(14.9)
Nationality	Japanese	1,462(98.6)	1,650(98.1)
	Foreign	21(1.4)	32(1.9)
Living alone	Yes	1,082(73.0)	1,186(70.5)
	No	401(27.0)	496(29.5)
Disability/ disease	Physical disability	140(9.4)	130(7.7)
	Mental disability	65(4.4)	55(3.3)
	Intellectual disability	2(0.1)	4(0.2)
	Psychiatric disorder	39(2.6)	87(5.2)
	Other physical diseases	325(21.9)	333(19.8)
	Alcoholic dependency	12(0.8)	2(0.1)
	None	900(60.7)	1,071(63.7)
History of hospitalization	Yes	77(5.2)	100(5.9)
	No	1,406(94.8)	1,582(94.1)
Long-term care status	Support need	74(5.0)	120(7.1)
	Long-term care need	255(17.2)	385(22.9)
	None	1,154(77.8)	1,177(70.0)
Working	Yes	138(9.3)	121(7.2)
	No	1,345(90.7)	1,561(92.8)
Previous use of public assistance	Yes	214(14.4)	240(14.3)
	No	1,269(85.6)	1,442(85.7)
Reasons for starting public assistance	Decreased income	762(51.4)	859(51.1)
	Disease	383(25.8)	368(21.9)
	Unemployment	131(8.8)	128(7.6)
	Divorce/bereavement	43(2.9)	61(3.6)
	Other reasons	164(11.1)	266(15.8)
History of facility admission	Yes	62(4.2)	97(5.8)
	No	1,421(95.8)	1,585(94.2)
Types of houses	Rental house	988(66.6)	912(54.2)
	Public house	353(23.8)	561(33.4)
	Own house	2(0.1)	5(0.3)
	Other houses	140(9.4)	204(12.1)
Working income^*^	Above median	34(2.3)	30(1.8)
	Below median	35(2.4)	31(1.8)
	None	1,414(95.3)	1,621(96.4)
Pension^†^	Above median	239(16.1)	416(24.7)
	Below median	241(16.3)	417(24.8)
	None	1,003(67.6)	849(50.5)

* The median monthly working income was 35,373 yen for male and 43,858 yen for female

† The median monthly pension was 61,670 yen for male and 45,708 yen for female

**Fig. 1:**
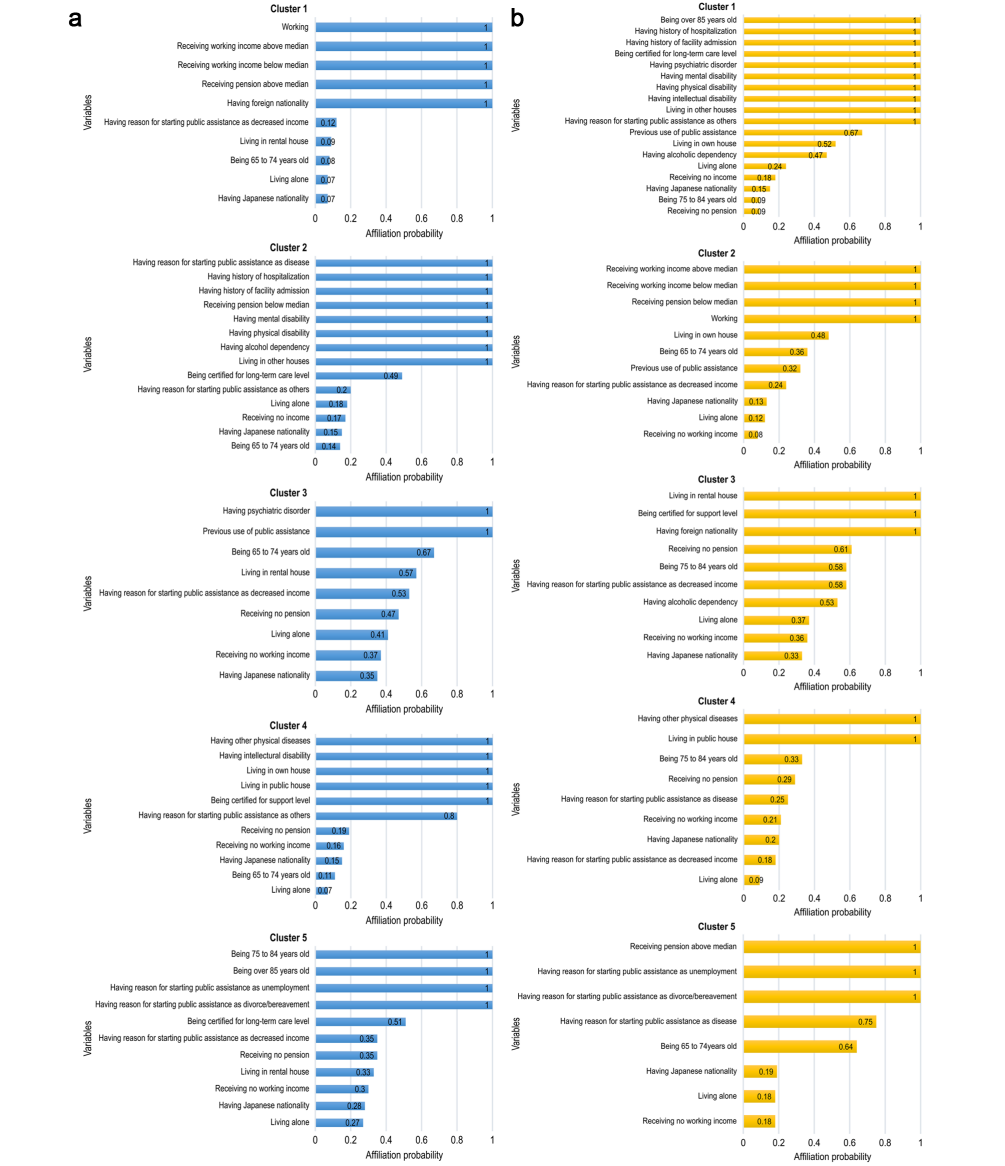
Affiliation probability of variables in the five-cluster model for males **(a)** and females **(b)**

Five-cluster model was identified conducting PLSA, after evaluating the criteria noted in the methods section (Additional file 3). We described the characteristics of clusters (Table 2a and b) using variables with the affiliation probability of 0.55 or higher (Fig. 1a and b). The characteristics of each cluster in both sexes were distinct. The clusters were named according to their characteristics. Four clusters were similar between sexes: (1) “workers” (male Cluster 1, female Cluster 2); (2) “people living at home with support need” (male Cluster 4, female Cluster 3); (3) “people who have started using public assistance due to life events” (male Cluster 5, female Cluster 5); and (4) “facility residents with disability” (male Cluster 2, female Cluster 1).

**Table 2:** Characteristics of the clusters in the five-cluster model for males (a) and females (b)

a		
Cluster	Name	Characteristics
1	Workers	People who work, earn working income, and receive pension above median (foreign nationals are included).
2	Facility residents with disability	People with mental or physical disabilities who have a history of hospitalization/facility admission, have reason for starting public assistance as disease, and live in other houses (people with alcoholic dependency are included).
3	People with psychiatric disorder living at home	People aged 65 to 74 years who live in rental house, have a psychiatric disorder/s, and have previously used public assistance.
4	People living at home with support need	People who live in public house or own house and are certified for support need (people with intellectual disability are included).
5	People who have started using public assistance due to life events	People aged over 75 years who have reason for starting public assistance as divorce/bereavement or unemployment.
**b**		
**Cluster**	**Name**	**Characteristics**
1	Facility residents aged over 85 years with disability/psychiatric disorder	People aged over 85 years who have a history of hospitalization/facility admission, previous use of public assistance, have psychiatric disorder/mental disability/physical disability, are certified for long-term care need, and live in other houses (people with intellectual disability are included).
2	Workers	People who work, earn income, and receive pension below median.
3	People living in rental house with support need	People aged 75 to 84 years who do not receive pension, live in rental house, are certified for support need, and have reason for starting public assistance as decreased income (foreign nationals are included).
4	People with physical disease living in public house	People who live in public house and have other physical disease.
5	People who have started using public assistance due to life events	People aged 65 to 74 years who receive pension above median and have reason for starting public assistance as divorce/bereavement, unemployment, or disease.

### Qualitative findings

Four caseworkers (all male) participated in the interviews. Their years of experience as caseworkers ranged from 3 to 9 years with an average of 5.5 years. The average duration of a joint interview was 82.5 min.

All caseworkers recalled older recipients with similar characteristics in female Cluster 1, 2, and 4. Some caseworkers did so in male Cluster 1, 2, and 3, and female Cluster 3 and 5. They described additional attributes of older recipients based on the characteristics of these clusters (Table 3a and b).

**Table 3:** Additional attributes of older public assistance recipients described by caseworkers from the characteristics of the clusters for males (a) and females (b)

a		
Cluster	Characteristics of cluster from quantitative results	Additional attributes of older recipients described by caseworkers
1	People who work, earn working income, and receive pension above median (foreign nationals are included).	Active seniors People receiving low levels of livelihood assistance
2	People with mental or physical disabilities who have a history of hospitalization/facility admission, have reason for starting public assistance as disease, and live in other houses (people with alcoholic dependency are included).	People who reside in long-term care health facilities after hospitalization or in relief facilities People having mental disability and no permanent residence People who have psychiatric disorder and reside in special nursing homes
3	People aged 65 to 74 years who live in rental house, have a psychiatric disorder/s, and have previously used public assistance.	People aged 75 and over
4	People who live in public house or own house and are certified for support need (people with intellectual disability are included).	(All caseworkers said that they could not think of any older recipients from this cluster.)
5	People aged over 75 years who have reason for starting public assistance as divorce/bereavement or unemployment.	(All caseworkers said that they could not think of any older recipients from this cluster.)
**b**		
**Cluster**	**Characteristics of cluster from quantitative findings**	**Additional attributes of older recipients described by caseworkers**
1	People aged over 85 years who have a history of hospitalization/facility admission, previous use of public assistance, have psychiatric disorder/mental disability/physical disability, are certified for long-term care need, and live in other houses (people with intellectual disability are included).	People residing in private nursing homes or serviced housing for older people People residing in certain facilities because of the decline in their functional abilities People residing in special nursing homes
2	People who work, earn income, and receive pension below median.	People who are motivated to work People regularly visiting doctors and reporting their incomes People who are independent and energetic in their daily activities People working as janitors or cooking assistants and seeking orthopedic treatment for back pain
3	People aged 75 to 84 years who do not receive pension, live in rental house, are certified for support need, and have reason for starting public assistance as decreased income (foreign nationals are included).	Healthy and energetic people People with dementia who lack family support
4	People who live in public house and have other physical diseases.	People who are single People in female Cluster 3
5	People aged 65 to 74 years who receive pension above median and have reason for starting public assistance as divorce/bereavement, unemployment, or disease.	People in female Cluster 4

#### Additional attributes of older recipients described by caseworkers

The caseworkers indicated that older male recipients in Cluster 1 might be “active seniors” or “people receiving low levels of livelihood assistance.“

*Some of them are self-supporting to some extent.* (Caseworker 3, 3 years of experience, municipality B)

*Individuals who receive a pension or working income often mention that they have lower living expenses compared to others, resulting in lower levels of livelihood assistance.* (Caseworker 2, 9 years of experience, municipality A)

Male Cluster 2 of older recipients was characterized by individuals described as “residing in long-term care health facilities after hospitalization or in relief facilities,” “having mental disability and no permanent residence,” or “having psychiatric disorder and residing in special nursing homes.”

*I can think of people who went to long-term care health facilities after being in relief facilities or after being hospitalized.* (Caseworker 1, 7 years of experience, municipality A)

The caseworkers reported that they could also think of recipients aged 75 and older in Cluster 3 of male recipients.

Female Cluster 1 of older recipients was described as “residing in private nursing homes or serviced housing for older people,” “residing in certain facilities because of the decline in their functional abilities,” or “residing in special nursing homes.”

*I think it is natural that people who are 85 years or older are in declining physical condition and their ADLs (activities of daily living) are also declining, so I am aware that many of them reside in certain facilities.* (Caseworker 1, 7 years of experience, municipality A)

*I have the impression that they are grandmothers in special nursing homes.* (Caseworker 3, 3 years of experience, municipality B)

Caseworkers noted that from female Cluster 2 of older recipients, they could identify individuals described as “motivated to work,“ “regularly visiting doctors and reporting their incomes,“ “independent and energetic in their daily activities,“ or “working as janitors or cooking assistants and seeking orthopedic treatment for back pain.“

*It seems to me that older female recipients who continue working display a relatively higher level of self-control and attention to detail. Many of them do not miss regular medical visits and file their income reports properly.* (Caseworker 1, 7 years of experience, municipality A)

*It immediately came to mind that one janitor or cooking assistant frequently went to the orthopedist due to back pain.* (Caseworker 3, 3 years of experience, municipality B)

Caseworkers mentioned that when recalling older recipients in female Cluster 3, they also observed individuals described as “healthy and energetic people” or “people with dementia who lack family support.“

*I advise those who are in good health and well-being to take care of their bodies, thus minimizing the need for nursing care.* (Caseworker 2, 9 years of experience, municipality A)

*As someone responsible for individuals with dementia, I can recall an old lady who has exhibited increased energy and confusion, and unfortunately lacks any relatives.* (Caseworker 3, 3 years of experience, municipality B)

Caseworkers observed an overlap of characteristics between older recipients in female Cluster 4 and those in female Cluster 3. A similar overlap of characteristics was also observed between older recipients in female Cluster 5 and those in female Cluster 4.

*In my opinion, the individuals in female Cluster 4 can be categorized within female Cluster 3. I perceive female Cluster 4 as a substantial group.* (Caseworker 1, 7 years of experience, municipality A)

*The individuals that come to mind belong to either female Cluster 4, female Cluster 5, or both.* (Caseworker 4, 3 years of experience, municipality B)

However, no additional attributes of older recipients were described in male Cluster 4 and 5 because none of them could think of any older recipients from these two clusters.

#### Reasons as to why caseworkers could not think of any older recipients from the cluster

The reasons as to why caseworkers could not think of any older recipients in the cluster are presented in Additional file 4: Table S2a and b. Three major themes emerged from the findings: (1) caseworkers did not pay attention to the variables used in this study when supporting older recipients; (2) the combination of variables in the same cluster was difficult to understand; and (3) caseworkers could not recognize older recipients with certain characteristics (Table 4).

**Table 4:** Summarizing emerging themes about reasons as to why caseworkers could not think of any older recipients from the cluster

Emerging themes	Summary of responses
Caseworkers did not pay attention to the variables used in this study when supporting older recipients.	The caseworker stated that he did not pay attention to how much pension older recipients received (in male Cluster 1): “Of course, I do not distinguish between providing support to older recipients with and without pension.” (Caseworker 1, 7 years of experience, municipality A) One of the caseworkers also indicated that he did not pay attention to information on psychiatric disorders among older recipients (in male Cluster 3): “I think their symptoms of psychiatric disorder weaken gradually as they get older. I would rather need information about their functional ability.” (Caseworker 1, 7 years of experience, municipality A)
The combination of variables in the same cluster was difficult to understand.	In general, the caseworkers expressed difficulty in understanding the cluster characteristics when older recipients’ reason for starting public assistance and their current age were in the same cluster (in male Cluster 5 and female Cluster 5), for example, “I think there are a lot of circumstances depending on the age when older recipients started receiving public assistance. So, it is difficult for me to understand the cluster characteristics that mix up their current age and the time when they started receiving public assistance.” (Caseworker 2, 9 years of experience, municipality A) In addition, the caseworkers felt that it was difficult to understand the cluster characteristics when various reasons for starting public assistance were in the same cluster (in male Cluster 5 and female Cluster 5), for example, “Some older recipients have received public assistance because they had trouble making ends meet before becoming old. Others have received public assistance because they quit their jobs when getting old and they could not live on their pension. I don’t think we can put all these people together.” (Caseworker 1, 7 years of experience, municipality A)
Caseworkers could not recognize older recipients with certain characteristics.	The caseworkers stated that they could not think of older recipients with mental or physical disability (in male Cluster 2), who had been certified for support need (in male Cluster 4 and female Cluster 3), who had started public assistance due to divorce/bereavement, unemployment, or disease (in female Cluster 5), and who received pension above median (in female Cluster 5). Some caseworkers suggested that older recipients who had been certified for support or long-term care need received more support from care managers than from caseworkers, therefore they could not think of older recipients: “I don’t really get involved with older recipients who are certified for support need.” (Caseworker 4, 3 years of experience, municipality B) “I receive inquiries from care managers about various things, and then I get involved (with them).” (Caseworker 2, 9 years of experience, municipality A) One of the caseworkers gave a detailed reason as to why he could not think of female older recipients who received pension above median: “For example, if their reason (for starting public assistance) is due to divorce or bereavement, I can think of a few older recipients who had relied on her husband’s income or pension, hadn’t paid their premiums, and then ended up receiving a small pension after their husband passed away.” (Caseworker 3, 3 years of experience, municipality B) Another caseworker expressed that he could think of female older recipients, but not male ones (in male Cluster 5): “I think of a couple in this cluster.” “I can easily think of female recipients.” (Caseworker 2, 9 years of experience, municipality A)

### Mixed methods findings

The integrated results showed concordance in female Cluster 1, 2, and 4; partial concordance in male Cluster 1, 2, and 3, and female Cluster 3 and 5; and discordance in male Cluster 4 and 5 (Additional file 5: Table S3a and b) [33].

## Discussion

Our soft clustering analysis found five distinct clusters (hereafter referred to as segments) of older recipients by sex. From the interviews, we have the evidence that several of these segments reflected older recipients in practice. We named them based on the key sociodemographic characteristics of each segment, which included “facility residents aged over 85 years with disability/psychiatric disorder”(Cluster 1), “workers”(Cluster 2), “people living in rental house with support need”(Cluster 3), “people with physical disease living in public house” (Cluster 4), and “people who have started using public assistance due to life events” (Cluster 5) in women, and “workers”(Cluster 1), “facility residents with disability”(Cluster 2), “people with psychiatric disorder living at home”(Cluster 3) in men. Caseworkers at municipality welfare offices recognized them as meaningful, recalling older recipients having those characteristics in their routine activities. Meanwhile, the soft clustering analysis also extracted some segments from which caseworkers did not think of older recipients in practice, including “people living at home with support need” (Cluster 4) and “people who have started using public assistance due to life events” (Cluster 5) in men.

In marketing, it is important that segments should “make sense” for stakeholders in terms of their utility and applicability [14]. On the one hand, in this study, the soft clustering technique successfully identified some segments that made sense for caseworkers. Notably, this study identified two of the female segments from which all of interviewed caseworkers could think of specific older recipients. They recalled older recipients who had lived in residences for older adults from the characteristics of female Cluster 1 (facility residents aged over 85 years with disability/psychiatric disorder). The recent governmental statistics have shown the increasing trends of older households on public assistance recipients residing in care facilities [9]; thus, older recipients in female Cluster 1 are those who caseworkers are more likely to meet in daily consultation. Interviewed caseworkers connected older recipients in female Cluster 2 (workers) with those who were financially independent by working and gained independence in daily life. Caseworkers receive an income declaration form at least every month or every 3 months from recipients who are capable to work; therefore, they can recall these older recipients fairly easily [35].

On the other hand, there were several segments from which caseworkers did not think of older recipients in practice. Some reported that they did not recall older recipients with certain characteristics (variables) because these variables did not help them conduct assistance activities. They also reported that the combination of variables in the same segment, that is, “the reason for starting public assistance and the current age” (male Cluster 5 and female Cluster 5) and “various reasons for starting public assistance” (male Cluster 5 and female Cluster 5), was difficult for them to understand. However, this finding raises the possibility that machine learning technique can potentially discover novel insights that are not obvious to caseworkers [16, 36]. Another reason as to why caseworkers could not think of any older recipients from the segment was that they could not recognize older recipients with mental or physical disability in combination with other segment characteristics (in male Cluster 2), who had been certified for support need (in male Cluster 4 and female Cluster 3), who had started public assistance due to as divorce/bereavement, unemployment, or disease (in female Cluster 5), and who received pension above median (in female Cluster 5). Older recipients who caseworkers have met are more likely to depend on their casework experience and role in support activities. In this study, a few caseworkers faced difficulties in recalling older male recipients who had mental or physical disabilities in combination with other segment characteristics, as their representation was relatively low (mental disability:4.4%; physical disability:9.4%). Furthermore, caseworkers tend to have such a heavy workload that they may not be able to remember every recipient information. The number of households that each caseworker was in charge of was 96 in municipality A and 100 in municipality B. The previous study suggested that caseworkers had no time to reflect on support activities and had difficulty in establishing a relationship with recipients when the number of households per caseworker exceeded 90 [37]. Thus, again, we can consider using machine learning technique to obtain the segments that are otherwise difficult to find based on their personal experience [16, 36].

### Limitations

This study has several limitations. First, we used only older recipients’ sociodemographic and health-related variables. This limited us to capture detailed characteristics related to their daily behaviors, personality, social relationships, and socioeconomic status. However, the success of identifying some segments with limited variables may support the potential benefits of applying this method for a database with more rich information of older recipients, which warrants further study. Second, although the interviewees in the qualitative phase were experienced caseworkers from the welfare office that provided the data for the quantitative study, the number of our interviewees may be limited to cover the variability in their characteristics and experiences. Future studies can cover various types of caseworkers based on the factors such as years of casework, gender, and the area of workplace. Lastly, given the finding that none of the caseworkers could think of older recipients in some segments, the optimal number of segments (five-cluster model) may not be appropriate. In future studies, we need to consider determining the optimal number of segments among several candidates with caseworkers in order to create the segments that would make sense for them [14, 19].

### Practice and policy implications

Our results have important policy implications. According to the qualitative findings, the interviewees stated that the characteristics of older recipients in female Cluster 4 overlapped with that in female Cluster 3, and the characteristics of older recipients in female Cluster 5 overlapped with that in female Cluster 4. This finding demonstrates the advantage of the soft clustering method, which allows individuals to belong to multiple segments simultaneously, with specific probabilities to a particular segment [17, 18]. This also suggests that interventions based on segmentation using soft clustering can offer more flexible options to care providers. Thus, if the intervention designed for one segment of older recipients is not effective, providers can choose an alternative intervention for another segment they are most likely to belong to [16]. This is in contrast to interventions based on segmentation using hard clustering, in which individuals can only belong to a single segment [17]. The approach based on segmentation using hard clustering fails if the intervention for the selected segment is not effective for older recipients in that segment. Moreover, soft clustering method in our study could identify segments that reflected older recipients in practice. This finding indicates that this approach enables care providers to plan effective interventions for the resulting segments. In Japan, each welfare office needs to analyze data of public assistance recipients and prioritize the intervention targets in the health management support program for public assistance recipients [11, 12]. For example, interventions such as mental health support program may be prioritized for the segment of “people with psychiatric disorder living at home” (male Cluster 3), while support for making regular visits to the doctor may be effective for the segment “people with physical disease living in public house” (female Cluster 4). Caseworkers and healthcare providers in welfare offices can utilize the identified segments for examining their association with health status or health behavior and then recommend tailored intervention for each segment according to the results. With this approach, inexperienced caseworkers, or those unfamiliar with health support, can prioritize recipients with the greatest need for support interventions, such as health check-ups and referrals to health care professionals and community sectors.

## Conclusions

A soft clustering method in our study identified five distinct segments of older public assistance recipients by sex. Employing a clustering technique can prove valuable in identifying meaningful segments among older recipients, potentially unveiling previously unrecognized segments that may not emerge through regular consultation practices followed by caseworkers. Segmentation using a clustering method can be a promising strategy for prioritizing the intervention targets and planning support interventions tailored to different needs of public assistance recipients.

### Electronic supplementary material

Below is the link to the electronic supplementary material.

Additional file 1: Table S1. List of variables used in the quantitative analysis.

Additional file 2: Interview guide.

Additional file 3: Determining the optimal number of clusters. Figure S1. AIC and BIC scores from two to ten patterns of clusters for males (a) and females (b). Figure S2. The number of people in each cluster from two to ten patterns of clusters for males (a) and females (b). Figure S3. Affiliation probability of variables in the four-cluster model for males (a) and females (b).

Additional file 4: Table S2. Reasons as to why caseworkers could not think of any older public assistance recipients from the clusters for males (a) and females (b).

Additional file 5: Table S3. Joint display of quantitative and qualitative findings regarding the five-cluster model for male (a) and female (b) older public assistance recipients.

## Data Availability

The data that support the findings of this study are available from the participating municipalities in Japan. However, restrictions apply to the availability of these data, which were used under licence for the current study, and so are not publicly available. Data are available from the authors upon reasonable request and with permission of the participating municipalities.

## Abbreviations

PLSA: Probabilistic latent semantic analysis
AIC: Akaike information criterion
BIC: Bayesian information criterion
