# Supplementary material for: Identifying meaningful subpopulation segments among older public assistance recipients: a mixed methods study to develop tailor-made health and welfare interventions
Source: Int J Equity Health. 2023 Aug 3;22:146. doi: 10.1186/s12939-023-01959-7 (PMC10401839; doi:10.1186/s12939-023-01959-7)
Supplement: Supplementary file 1 — Additional file 1: Table S1. List of variables used in the quantitative analysis. [file 12939_2023_1959_MOESM1_ESM.docx]

**Additional file 1**

Table S1. List of variables used in the quantitative analysis

| **Sociodemographic variables** | |
| --- | --- |
| **Name of variables** | **Variables used in the analysis** |
| Age | 65 to 74 years old |
|  | 75 to 84 years old |
|  | 85 years old and over |
| Nationality | Japanese |
|  | Foreign |
| Living alone | Yes |
|  | No |
| Working | Yes |
|  | No |
| Previous use of public assistance | Yes |
|  | No |
| Reasons for starting public assistance | Decreased income |
|  | Diseases |
|  | Unemployment |
|  | Divorce/bereavement |
|  | Other reasons |
| History of facility admission | Yes |
|  | No |
| Types of houses | Rental house |
|  | Public house |
|  | Own house |
|  | Other houses |
| Working income | Above median |
|  | Below median |
|  | None |
| Pension | Above median |
|  | Below median |
|  | None |

| **Health-related variables** | |
| --- | --- |
| Disabilities/diseases* | Physical disability |
|  | Mental disability |
|  | Intellectual disability |
|  | Psychiatric disorder |
|  | Other physical diseases |
|  | Alcoholic dependency |
|  | None |
| History of hospitalization^†^ | Yes |
|  | No |
| Long-term care status^‡^ | Support need |
|  | Long-term care need |
|  | None |

* Information on disabilities/diseases was obtained from staff members in the welfare offices of each municipality when making a decision about an application for public assistance. People with physical disability are those who suffer from visual impairment, hearing impairment, limb disability, organ dysfunction, and others; people with mental disability are those who suffer from chronic mental disorders such as schizophrenia, mood disorder, and epilepsy; and people with intellectual disability are those who interfere with their daily lives due to disability. For those with these disabilities, a certificate of disability is issued to facilitate access to welfare services [1].

† A history of hospitalization was obtained by staff members in the welfare offices of each municipality when making a decision about an application for public assistance. This is information regarding whether the public assistance recipient has ever been hospitalized in a medical institution.

‡In the Japanese public long-term care insurance system, there are seven nationally standardized levels of long-term care need (requiring support: levels 1 and 2, and requiring long-term care need: levels 1–5).

For this insurance benefit, applicants need to be 65 years or older, who are potentially in need of long-term care, and 40 years or older, with the designated diseases. Insurance benefits are evaluated and certified by insurers (municipal governments) based on the level of long-term care need [2].

**Reference**

1. 3-2 Welfare of persons with disabilities. In Trends in national welfare and long-term care (in Japanese). Journal of Health and Welfare Statistics. 2019; 66(10):132-141.

2. Sakamoto H, Rahman M, Nomura S, et al. Japan health system review [online]. World Health Organization. Regional Office for South-East Asia, 2018. <https://apps.who.int/iris/handle/10665/259941>. Accessed June 10 2023.
