# Supplementary material for: Identifying meaningful subpopulation segments among older public assistance recipients: a mixed methods study to develop tailor-made health and welfare interventions
Source: Int J Equity Health. 2023 Aug 3;22:146. doi: 10.1186/s12939-023-01959-7 (PMC10401839; doi:10.1186/s12939-023-01959-7)
Supplement: Supplementary file 2 — Additional file 2: Interview guide. [file 12939_2023_1959_MOESM2_ESM.docx]

**Additional file 2**

Interview guide

**Introduction**

1. How many years have you been engaged in public assistance casework?

2. What is your current position in the welfare office?

**The main part**

3. There are five clusters of male older public assistance recipients as shown in the table.

Can you think of any older recipient(s) who has(have) similar characteristics in the cluster? Please provide their detailed characteristics.

4. (If interviewees note that they can think of older recipient(s) who is(are) slightly different from older recipients in the cluster) Can you indicate their characteristics that are different from those in the cluster?

5. (If interviewees note that they cannot think of any older recipients that fit the characteristics of the cluster) Can you tell me the reason why you cannot think of any older recipients?

Interviewees are asked questions 3 to 5 about each cluster of male older recipients shown in Figure 1a and Table 2a and that of female older recipients shown in Figure 1b and Table 2b.
