# Supplementary material for: Identifying meaningful subpopulation segments among older public assistance recipients: a mixed methods study to develop tailor-made health and welfare interventions
Source: Int J Equity Health. 2023 Aug 3;22:146. doi: 10.1186/s12939-023-01959-7 (PMC10401839; doi:10.1186/s12939-023-01959-7)
Supplement: Supplementary file 3 — Additional file 3: Determining the optimal number of clusters. Figure S1. AIC and BIC scores from two to ten patterns of clusters for males (a) and females (b). Figure S2. The number of people in each cluster from two to ten patterns of clusters for males (a) and females (b). Figure S3. Affiliation probability of variables in the four-cluster model for males (a) and females (b). [file 12939_2023_1959_MOESM3_ESM.docx]

**Additional file 3**

Determining the optimal number of clusters

We determined the optimal number of clusters in the following steps.

**1. AIC and BIC**

The two-cluster model had the lowest values of Akaike information criterion (AIC) and Bayesian information criterion (BIC), and was found out be the preferred model according to these criteria (Figure S1a and b). However, we could not describe the characteristics of each cluster because the variables belonged to only either one of the two clusters. Therefore, we determined that two-cluster model was not optimal.

**2. Size of clusters**

The number of people in each cluster was evaluated. When the number of clusters for men was six or more and the number of clusters for women was seven or more, some clusters had an extremely low number of people (Figure S2a and b). Thus, we determined that these cluster patterns were not optimal.

**3. Interpretability of clusters**

We investigated whether we could interpret the characteristics of each cluster in the three- to five-cluster model for males and females and the six-cluster model for females. It was difficult to interpret some clusters’ characteristics in the three-cluster model for males and females and the six-cluster model for females. The four- (Figure S3a and b) and five-cluster models were candidates for the optimal number of clusters for both males and females. After discussions among the authors, the five-cluster model was considered to be optimal for both sexes.

a


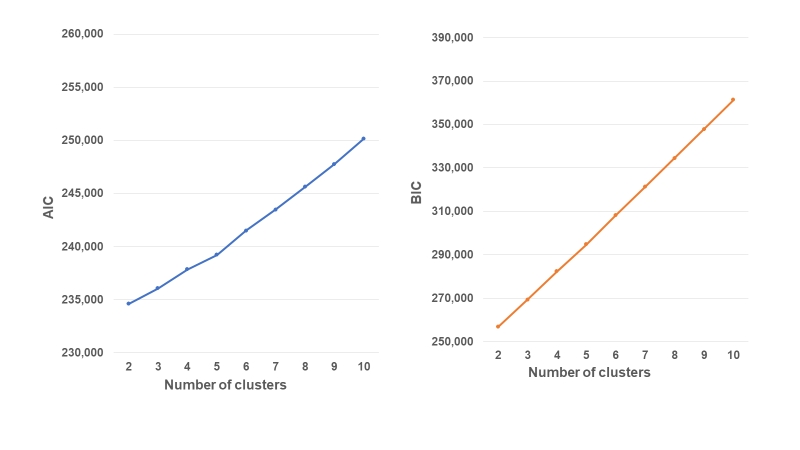


b


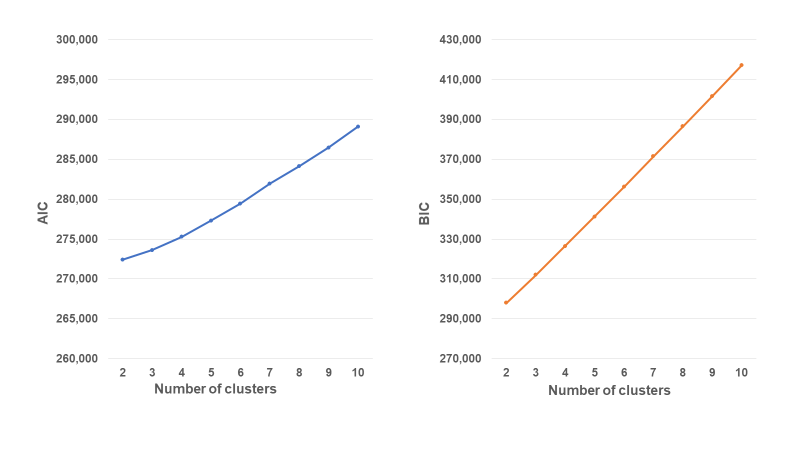


Figure S1. AIC and BIC scores from two to ten patterns of clusters for males (a) and females (b)

AIC: Akaike information criterion, BIC: Bayesian information criterion

a


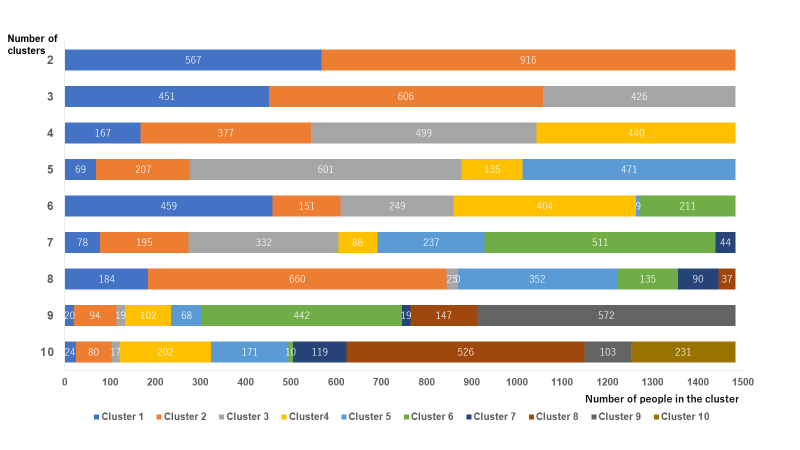


b


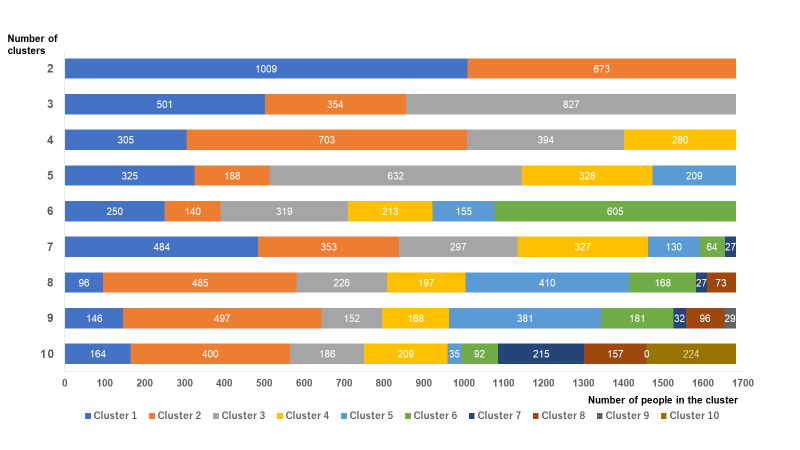


Figure S2. The number of people in each cluster from two to ten patterns of clusters for males (a) and females (b)

a


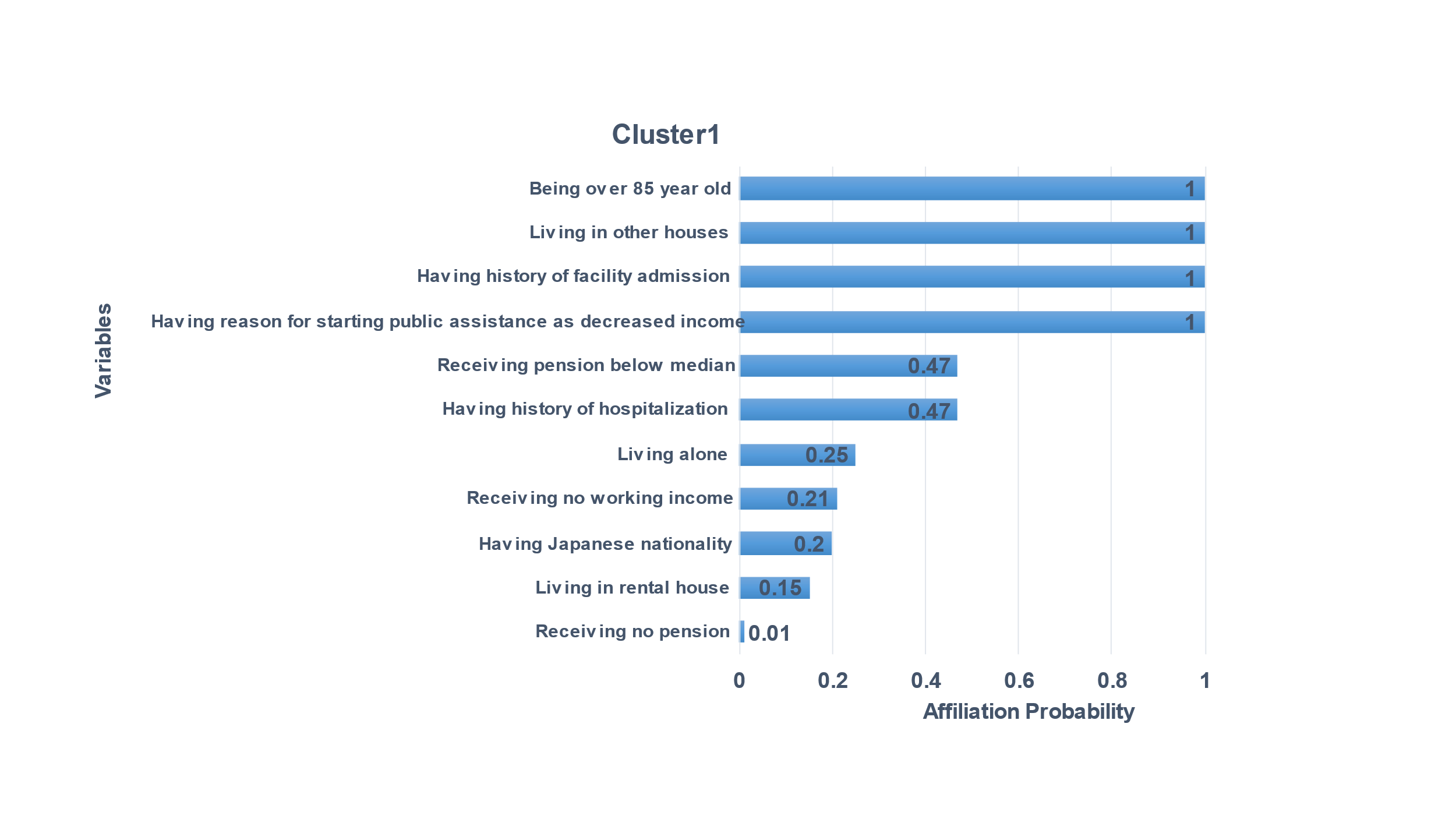


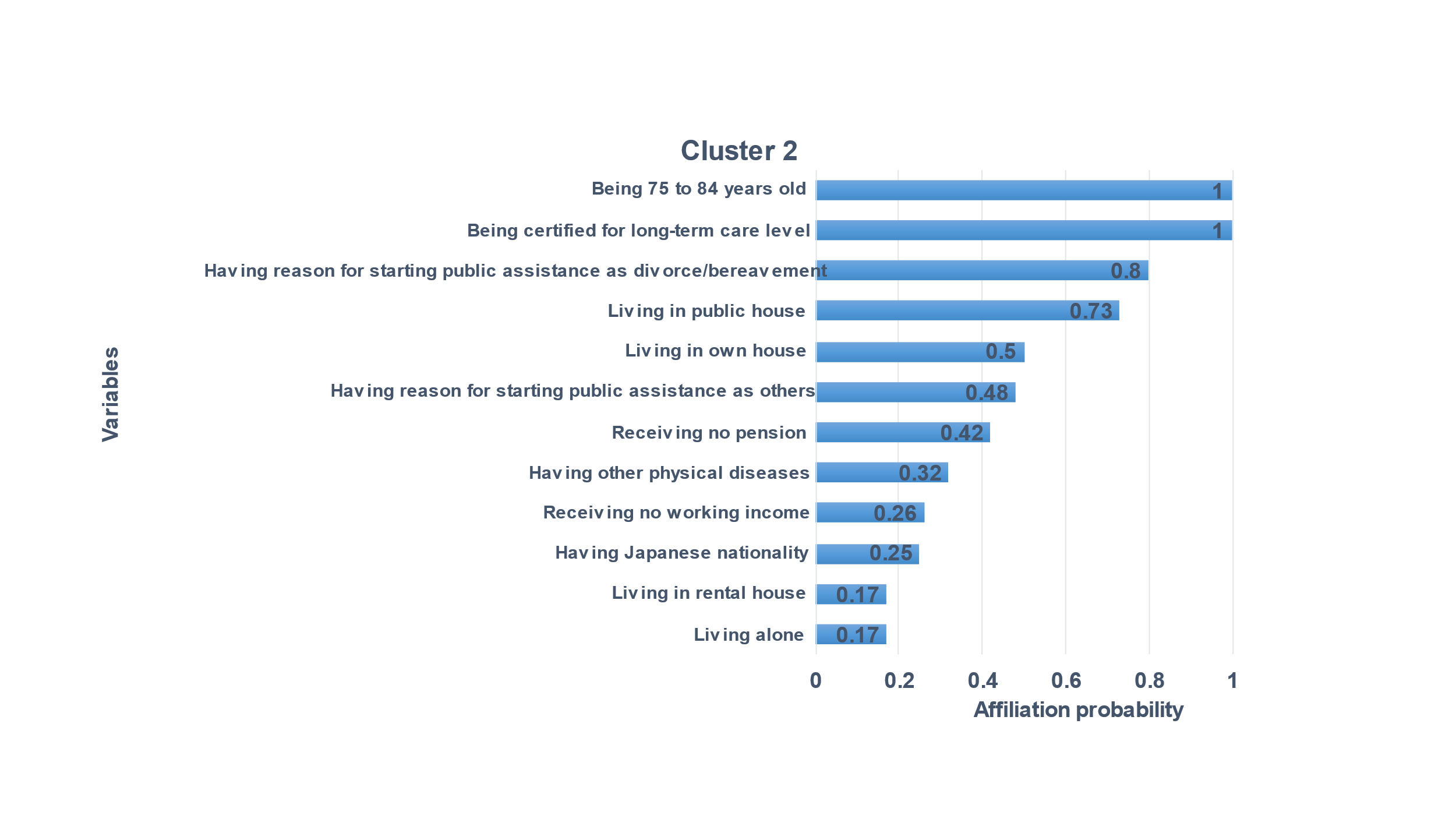


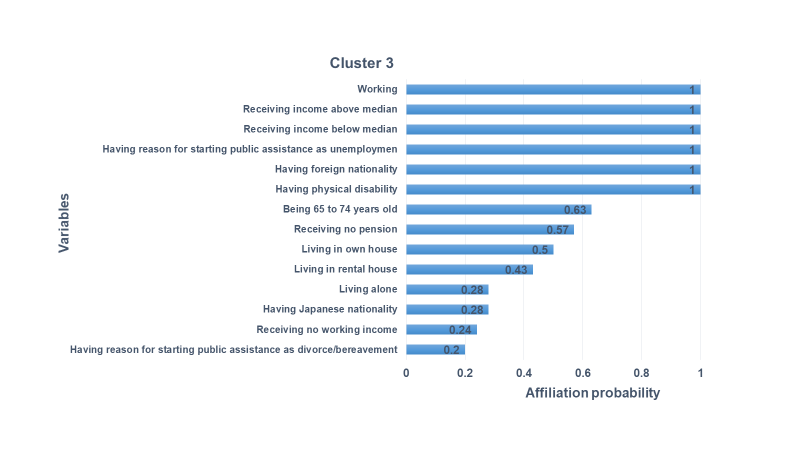


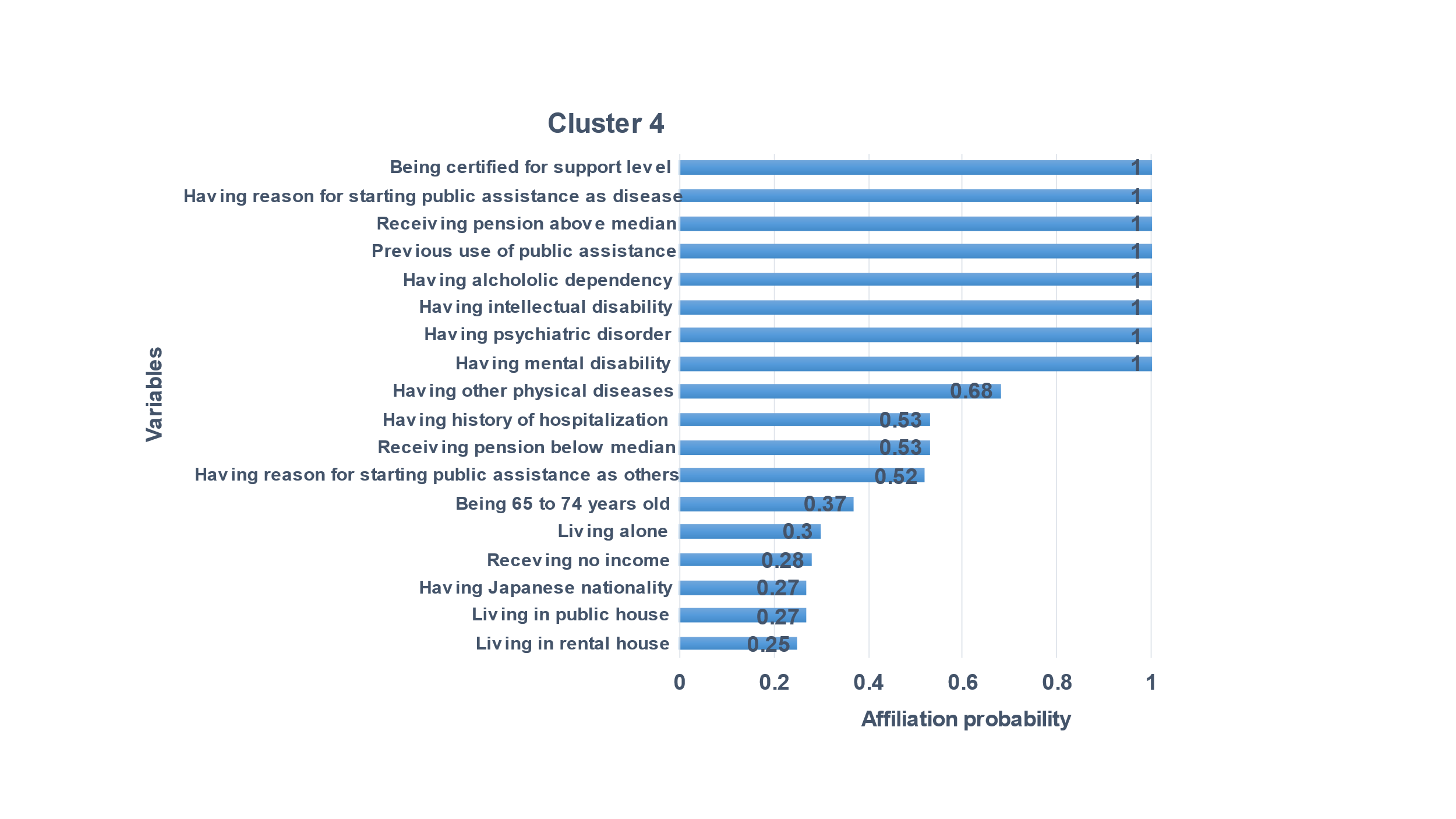


b


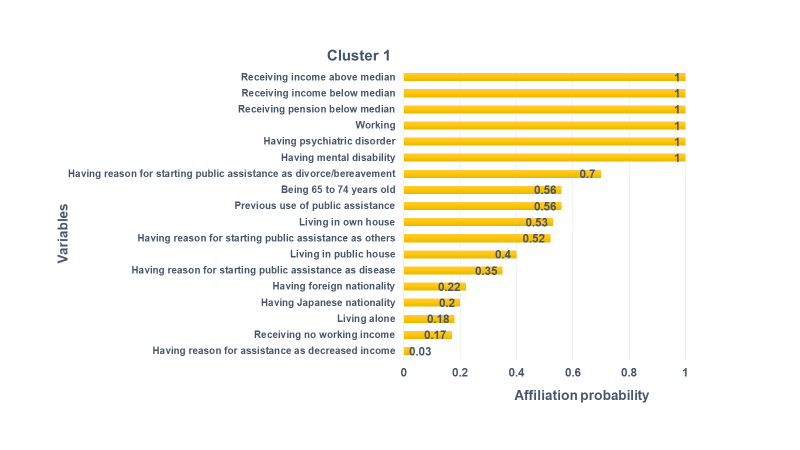


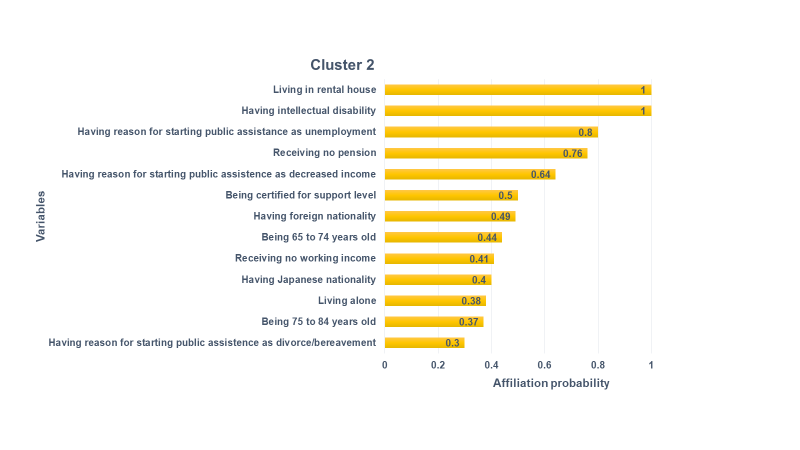


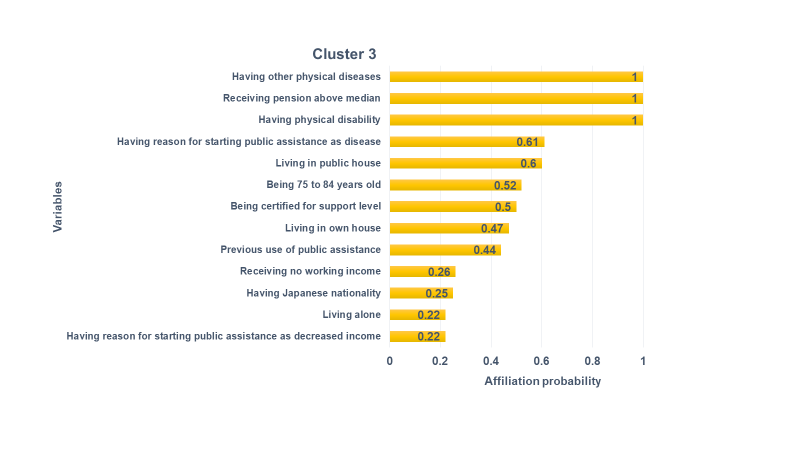


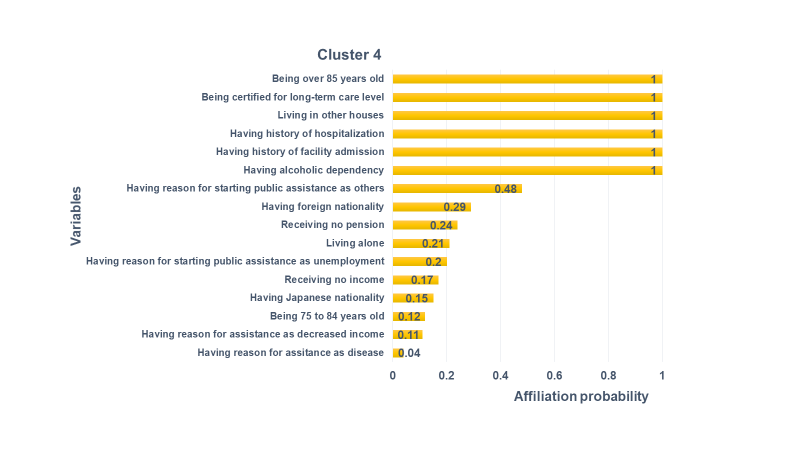


Figure S3. Affiliation probability of variables in the four-cluster model for males (a) and females (b)
