# Supplementary material for: Identifying meaningful subpopulation segments among older public assistance recipients: a mixed methods study to develop tailor-made health and welfare interventions
Source: Int J Equity Health. 2023 Aug 3;22:146. doi: 10.1186/s12939-023-01959-7 (PMC10401839; doi:10.1186/s12939-023-01959-7)
Supplement: Supplementary file 4 — Additional file 4: Table S2. Reasons as to why caseworkers could not think of any older public assistance recipients from the clusters for males (a) and females (b). [file 12939_2023_1959_MOESM4_ESM.docx]

**Additional file 4**

Table S2. Reasons as to why caseworkers could not think of any older public assistance recipients from the clusters for males (a) and females (b)

a

| Cluster | Characteristics of cluster  from quantitative findings | Reasons as to why caseworkers could not think of  any older recipients from the cluster | |
| --- | --- | --- | --- |
| 1 | People who work, earn working income, and receive pension above median (foreign nationals are included). | ⬝ I do not pay attention to how much pension older recipients receive. |  |
| 2 | People with mental or physical disabilities who have a history of hospitalization /facility admission, have reason for starting public assistance as disease, and live in other houses  (people with alcoholic dependency are included). | ⬝ I cannot think of older recipients with mental or physical  disability. |  |
| 3 | People aged 65 to 74 years who live in rental house, have a psychiatric disorder/s, and have previously used public assistance. | ⬝ I do not pay attention to information on psychiatric disorder among older recipients. |  |
| 4 | People who live in public house or own house and are certified for support need  (people with intellectual disability are included). | ⬝ I cannot think of older recipients who have been certified  for support need level. |  |
| 5 | People aged over 75 years who have reason for starting public assistance as divorce/bereavement or unemployment. | ⬝ I can think of female older recipients, but not male ones.  ⬝ It is difficult to understand the characteristics of the  cluster when the reason for starting public assistance and  their current age are in the same cluster.  ⬝ It is difficult to understand the characteristics of the  cluster when the different reasons for starting public  assistance are in the same cluster. |  |

b

| Cluster | Characteristics of cluster  from quantitative findings | Reasons as to why caseworkers could not think of  any older recipients from the cluster | |
| --- | --- | --- | --- |
| 1 | People aged over 85 years who have a history of hospitalization/facility admission, previous use of public assistance, have psychiatric disorder/mental disability/physical disability, are certified for long-term care need, and live in other houses  (people with intellectual disability are included). | (All caseworkers said that they could think of an(some) older recipient(s) from this cluster). |  |
| 2 | People who work, earn income, and receive pension below median. | (All caseworkers said that they could think of an(some) older recipient(s) from this cluster). |  |
| 3 | People aged 75 to 84 years who do not receive pension, live in rental house, are certified for support need, and have reason for starting public assistance as decreased income  (foreign nationals are included). | ⬝ I cannot think of older recipients who have been certified  for support need level. |  |
| 4 | People who live in public house and have other physical diseases. | (All caseworkers said that they could think of an(some) older recipient(s) from this cluster). |  |
| 5 | People aged 65 to 74 years who receive pension above median and have reason for starting public assistance as divorce/bereavement, unemployment, or disease. | ⬝ I cannot think of older recipients who have started public  assistance for these reasons.  ⬝ I cannot think of any older recipients who receive  pension above median.  ⬝ It is difficult to understand the characteristics of the  cluster when the reason for starting public  assistance and their current age are in the same cluster.  ⬝ It is difficult to understand the characteristics of the  cluster when the different reasons for starting public assistance are in the same cluster. |  |
