# Supplementary material for: Identifying meaningful subpopulation segments among older public assistance recipients: a mixed methods study to develop tailor-made health and welfare interventions
Source: Int J Equity Health. 2023 Aug 3;22:146. doi: 10.1186/s12939-023-01959-7 (PMC10401839; doi:10.1186/s12939-023-01959-7)
Supplement: Supplementary file 5 — Additional file 5: Table S3. Joint display of quantitative and qualitative findings regarding the five-cluster model for male (a) and female (b) older public assistance recipients. [file 12939_2023_1959_MOESM5_ESM.docx]

**Additional file 5**

Table S3. Joint display of quantitative and qualitative findings regarding the five-cluster model for male (a) and female (b) older public assistance recipients

a

| Cluster | Quantitative findings | | Qualitative findings | | *Fit of QUAN and QUAL findings |
| --- | --- | --- | --- | --- | --- |
|  | Name | Characteristics | Additional attributes of older recipients  described by caseworkers | Reasons as to why caseworkers could not think of any older recipients from the cluster |  |
| 1 | Workers | People who work, earn working income, and receive pension above median  (foreign nationals are included). | ⬝ Active seniors  ⬝ People receiving low levels of livelihood assistance | ⬝ I do not pay attention to how much pension older recipients receive. | Partial concordance |
| 2 | Facility residents with disability | People with mental or physical disabilities who have a history of hospitalization /facility admission, have reason for starting public assistance as disease, and live in other houses  (people with alcoholic dependency are included). | ⬝ People who reside in long-term care health facilities after hospitalization or in relief facilities  ⬝ People having mental disability and no permanent residence  ⬝ People who have psychiatric disorder and reside in special nursing homes | ⬝ I cannot think of older recipients with mental or physical disability. | Partial concordance |
| 3 | People with psychiatric disorder living at home | People aged 65 to 74 years who live in rental house, have a psychiatric disorder/s, and have previously used public assistance. | ⬝ People aged 75 and over | ⬝ I do not pay attention to information on psychiatric disorder among older recipients. | Partial concordance |
| 4 | People living at home with support need | People who live in public house or own house and are certified for support need  (people with intellectual disability are included). | (All caseworkers said that they could not think  of any older recipients from this cluster). | ⬝ I cannot think of older recipients who have  been certified for support need level. | Discordance |
| 5 | People who have started using public assistance due to life events | People aged over 75 years who have reason for starting public assistance as divorce/bereavement or unemployment. | (All caseworkers said that they could not think  of any older recipients from this cluster). | ⬝ I can think of female older recipients, but  not male ones.  ⬝ It is difficult to understand the  characteristics of the cluster when  the reason for starting public assistance and  their current age is in the same cluster.  ⬝ It is difficult to understand the  characteristics of the cluster when the different reasons for starting public assistance are in the same cluster. | Discordance |

*QUAN: quantitative, QUAL: qualitative

b

| Cluster | Quantitative findings | | Qualitative findings | | *Fit of QUAN and QUAL findings |
| --- | --- | --- | --- | --- | --- |
|  | Name | Characteristics | Additional attributes of older recipients  described by caseworkers | Reasons as to why caseworkers could not think of any older recipients from the cluster |  |
| 1 | Facility residents aged over 85 years with disability/  psychiatric disorder | People aged over 85 years who have a history of hospitalization/facility admission, previous use of public assistance, have psychiatric disorder/mental disability/physical disability, are certified for long-term care need, and live in other houses  (people with intellectual disability are included). | ⬝ People residing in private nursing homes or serviced housing for older people  ⬝ People residing in certain facilities because of the decline in their functional abilities  ⬝ People residing in special nursing homes | (All caseworkers said that they could think of an (some) older recipient(s) from this cluster). | Concordance |
| 2 | Workers | People who work, earn income, and receive pension below median. | ⬝ People who are motivated to work  ⬝ People regularly visiting doctors and  reporting their incomes  ⬝ People who are independent and energetic in  their daily activities  ⬝ People working as janitors or cooking  assistants and seeking orthopedic treatment  for back pain | (All caseworkers said that they could think of  an (some) older recipient(s) from this cluster). | Concordance |
| 3 | People living in rental house with support need | People aged 75 to 84 years who do not receive pension, live in rental house, are certified for support need, and have reason for starting public assistance as decreased income  (foreign nationals are included). | ⬝ Healthy and energetic people  ⬝ People with dementia who lack family support | ⬝ I cannot think of any older recipients who have been certified for support need level. | Partial concordance |
| 4 | People with physical disease living in public house | People who live in public house and have other physical diseases. | ⬝ People who are single  ⬝ People in female Cluster 3 | (All caseworkers said that they could think of  an (some) older recipient(s) from this cluster). | Concordance |
| 5 | People who have started using public assistance due to life events | People aged 65 to 74 years who receive pension above median and have reason for starting public assistance as divorce/bereavement, unemployment, or disease. | ⬝ People in female Cluster 4 | ⬝ I cannot think of older recipients who have  started public assistance for these reasons.  ⬝ I cannot think of older recipients who  receive pension above median.  ⬝ It is difficult to understand the  characteristics of the cluster when the reason  for starting public assistance and their  current age is in the same cluster.  ⬝ It is difficult to understand the  characteristics of the cluster when the different reasons for starting public assistance are in the same cluster. | Partial concordance |

*QUAN: quantitative, QUAL: qualitative
